# Supplementary material for: O-GlcNAc and phosphorylation modifications on HtL1/FBA10 regulate wheat vernalization for flowering
Source: Nat Commun. 2025 Dec 27;17:999. doi: 10.1038/s41467-025-67734-0 (PMC12848038; doi:10.1038/s41467-025-67734-0)
Supplement: Supplementary file 1 — Supplementary Information [file 41467_2025_67734_MOESM1_ESM.pdf]

SUPPLEMENTARY INFORMATION

FOR

O-GlcNAc and phosphorylation modifications on HtL1/FBA10 regulate wheat vernalization for flowering

Pengfang Yang[1, 2, #], Yangyang Liu[1, 3, #], Qi Dong[1, 2, #], Yuting Miao[1, 2], Jianlong Zhang[1, 2], Shujuan Xu[1, 2, 4], Hong Zhao[1, 2], Yuda Niu[3, 5], Xueyong Zhang[6], Yunyuan Xu[3, 5], Zifeng Guo[1, 2, 3, ✉], Lijing Xing[3, 5, ✉], Kang Chong[1, 2, 3, 5, ✉]

[1]State Key Laboratory of Forage Breeding-by-Design and Utilization, Institute of Botany, Chinese Academy of Sciences, Beijing, 100093, China.

[2]University of Chinese Academy of Sciences, Beijing, 100049, China.

[3]China National Botanical Garden, Beijing, China.

[4]Institute of Science and Technology Austria, Klosterneuburg, 3400, Austria.

[5]Key Laboratory of Plant Molecular Physiology, Institute of Botany, Chinese Academy of Sciences, Beijing, 100093, China.

[6]Institute of Crop Sciences, Chinese Academy of Agricultural Sciences, Beijing, 100081, China.

[#] These authors contributed equally.

[✉]Corresponding author: Zifeng Guo, Lijing Xing, Kang Chong.

Email: guozifeng@ibcas.ac.cn; xinglijing@ibcas.ac.cn; chongk@ibcas.ac.cn

The PDF file includes:

Supplementary Figures 1-18
Supplementary Method

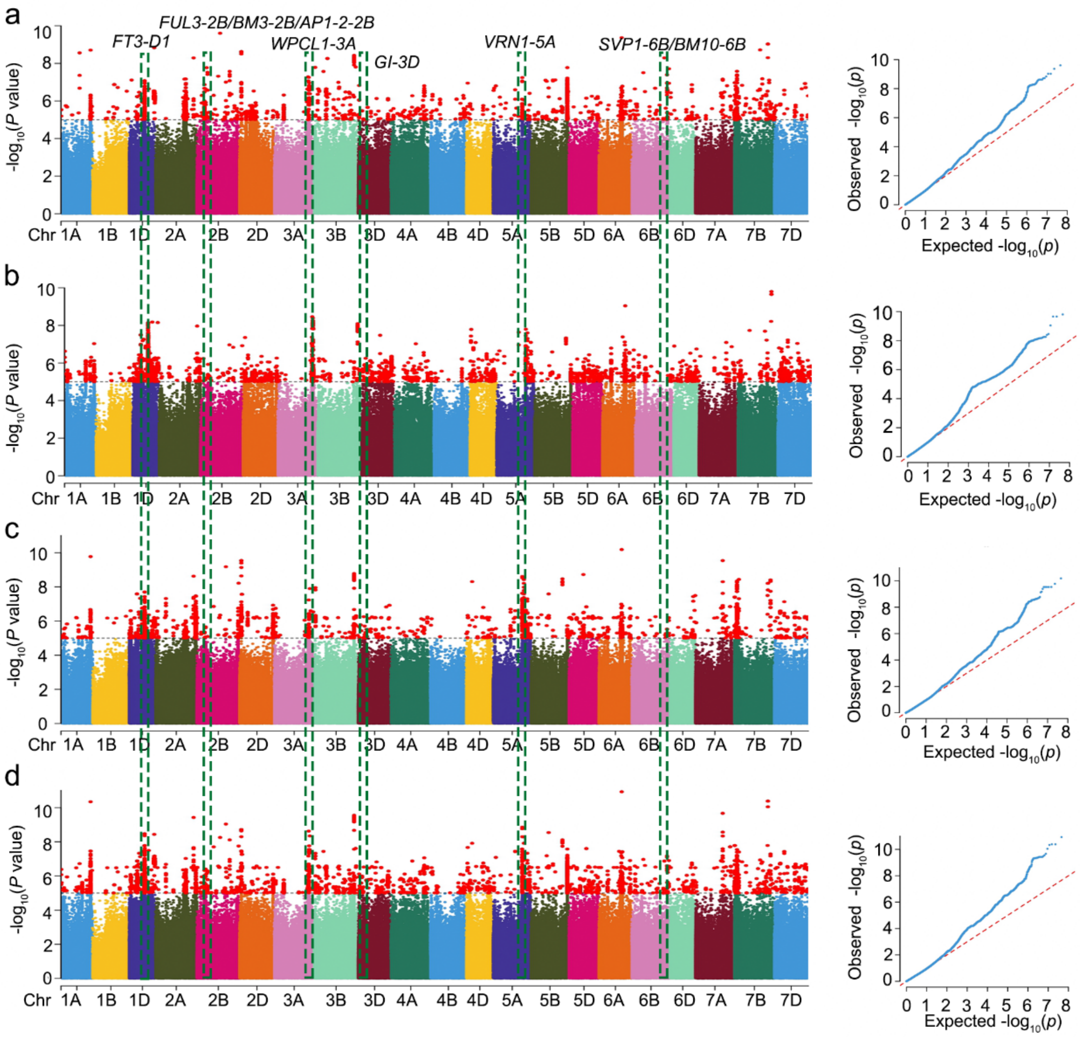

Supplementary Figure 1. GWAS analysis of wheat heading time in different years.

(a), **(b)**, and **(c)** The Manhattan plots (left) and QQ plots (right) for GWAS of heading time traits in 2014 **(a)**, 2015 **(b)**, and 2016 **(c)**. **(d)** The Manhattan plot (left) and QQ plot (right) from the GWAS analysis based on the Best Linear Unbiased Prediction (BLUP) values of phenotypes over the consecutive three years from 2014 to 2016.

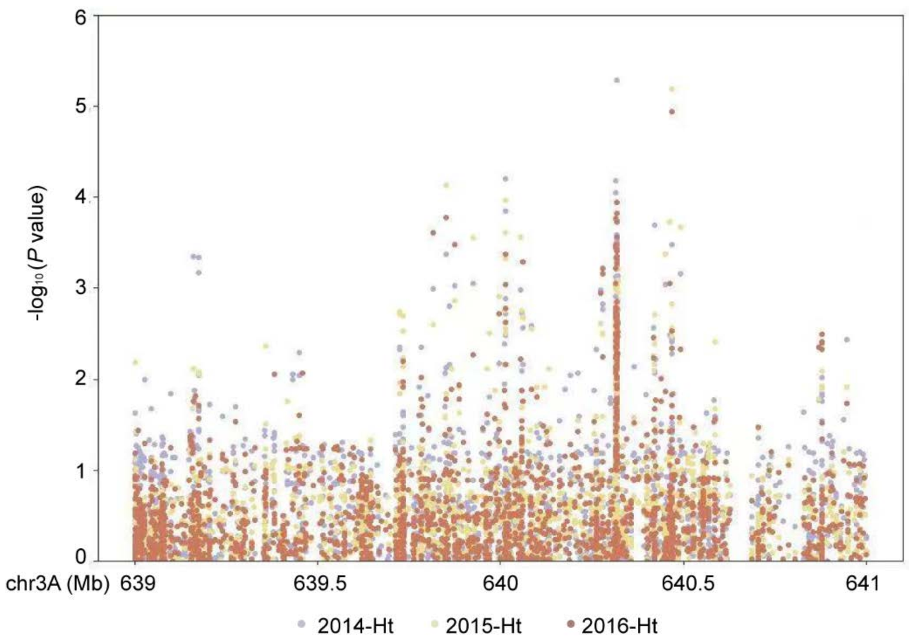

Supplementary Figure 2. GWAS analysis of wheat heading time in the range of 639 to 641 Mb on chromosome 3A.

The Manhattan plot of GWAS for heading time traits in the different years (2014-Ht: 2014-Heading time, 2015-Ht: 2015-Heading time, and 2016-Ht: 2016-Heading time) in the genomic range of 639 to 641 Mb on chromosome 3A.

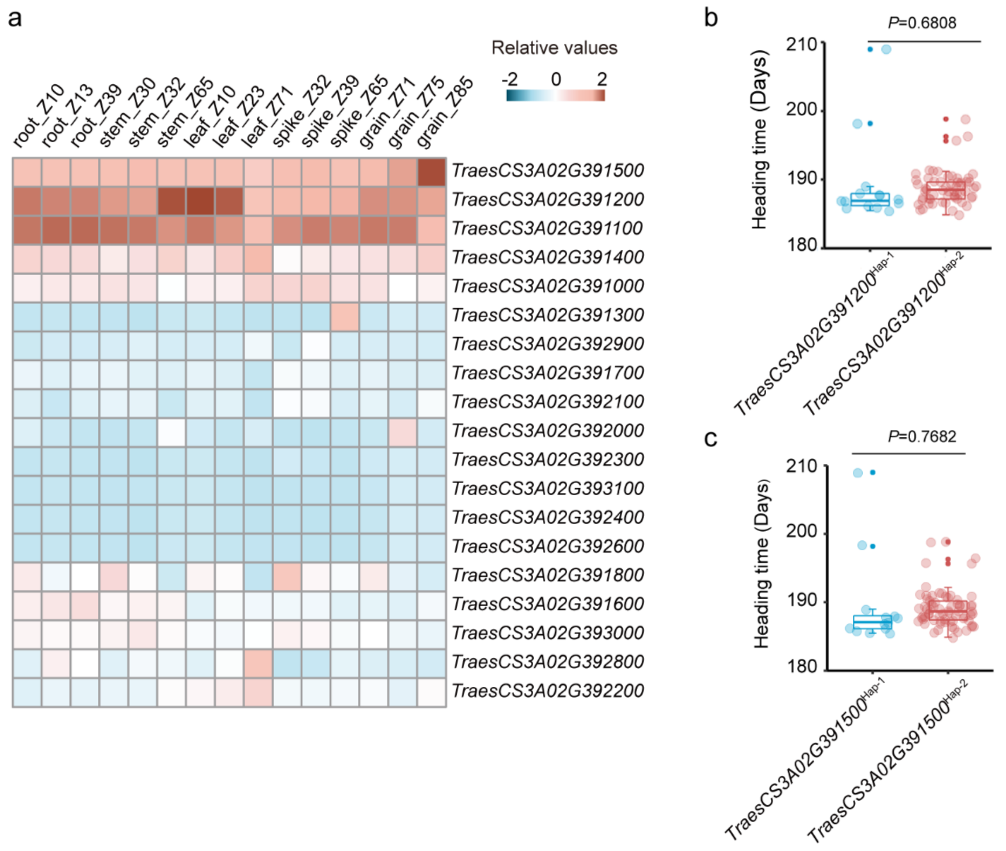

Supplementary Figure 3. Transcription and haplotype analyses of *TraesCS3A02G391100/-HtL1/FBA10*, *TraesCS3A02G391200* and *TraesCS3A02G391500/FBA11*.

(a) Tissue-specific expression profile of *TraesCS3A02G391100*/*HtL1*/*FBA10* and other candidate genes. Z: Zadoks decimal code, internationally used to represent developmental stages of cereal crops, with different numbers representing distinct developmental stages. The *TraesCS3A02G391900* was not detected in any wheat tissues, *TraesCS3A02G392500* and *TraesCS3A02G392700* exhibited extremely low or no expression across the tested tissues, thus they were not displayed in the figure. Colors represent relative expression levels, with blue and red indicating low and high expression levels, respectively. **(b)** Heading time analysis between two haplotypes of *TraesCS3A02G391200*$^{\text{Hap-1}}$ (n=14 accessions) and *TraesCS3A02G391200*$^{\text{Hap-2}}$ (n=58 accessions). **(c)** Heading time analysis between two haplotypes of *TraesCS3A02G391500*$^{\text{Hap-1}}$ (n=13 accessions) and *TraesCS3A02G391500*$^{\text{Hap-2}}$ (n=70 accessions). For each box, the upper and lower boundaries represent the 75th and 25th percentile, respectively. The middle horizontal lines represent the median. The whiskers represent 1.5 × the interquartile range. The individual data points are plotted as open circles. The solid dots beyond the whiskers represent outliers. Two-tailed Student's *t*-test was used for statistical analysis.

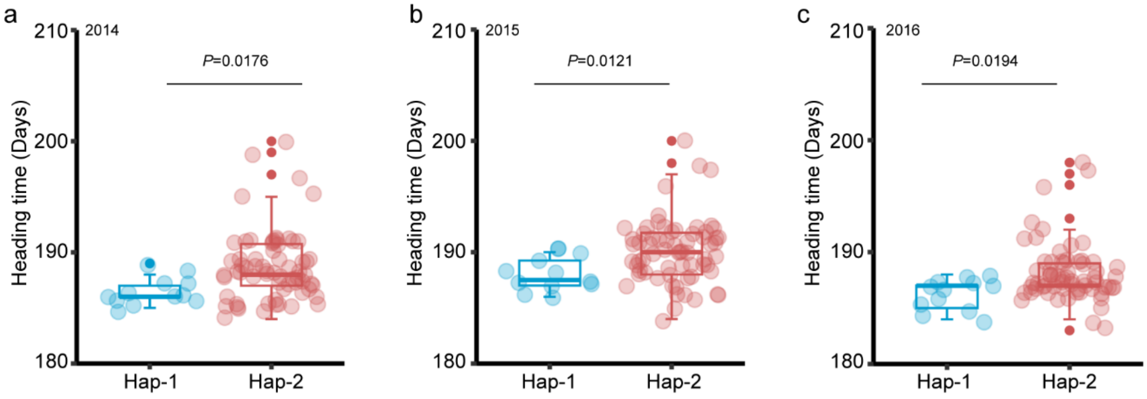

Supplementary Figure 4. The differences in heading time among materials containing two different haplotypes of *TraesCS3A02G391100/HtL1/FBA10*.

The differences in heading time among materials possessing two distinct haplotypes, Hap-1 (n=12 accessions) and Hap-2 (n=66 accessions) of *TraesCS3A02G391100/HtL1/FBA10* in 2014 **(a)** and 2015 **(b)**, and Hap-1 (n=12 accessions) and Hap-2 (n=65 accessions) of *TraesCS3A02G391100/-HtL1/FBA10* in 2016 **(c).** For each box, the upper and lower boundaries represent the 75th and 25th percentile, respectively. The middle horizontal lines represent the median. The whiskers represent 1.5 × the interquartile range. The individual data points are plotted as open circles. The solid dots beyond the whiskers represent outliers. Two-tailed Student's *t*-test was used for statistical analysis.

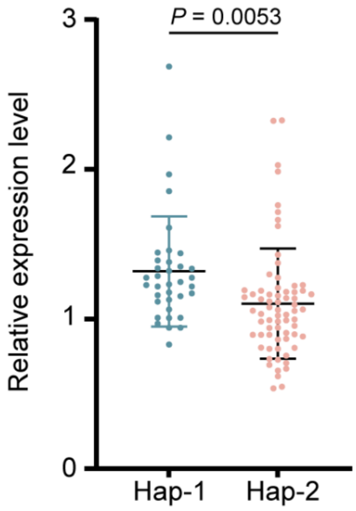

Supplementary Figure 5. Expression analysis of *HtL1/FBA10* in Hap-1 and Hap-2 groups.

The transcript levels of *HtL1/FBA10* in plumules of Hap-1 (n=12) and randomly selected Hap-2 (n=23) accessions were analyzed by RT–qPCR. Three independent biological replicates were conducted, and the data from all replicates are presented in the figure. Data are means $\pm$ SD, two-tailed Student's *t*-test was used to determine the *P* value.

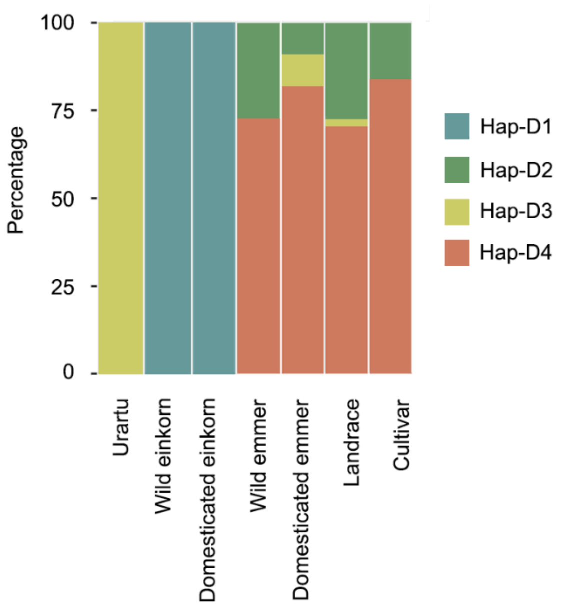

Supplementary Figure 6. The frequencies of *TraesCS3A02G391100/HtL1/FBA10* haplotypes across diploid, tetraploid, and heterohexaploid wheat accessions.

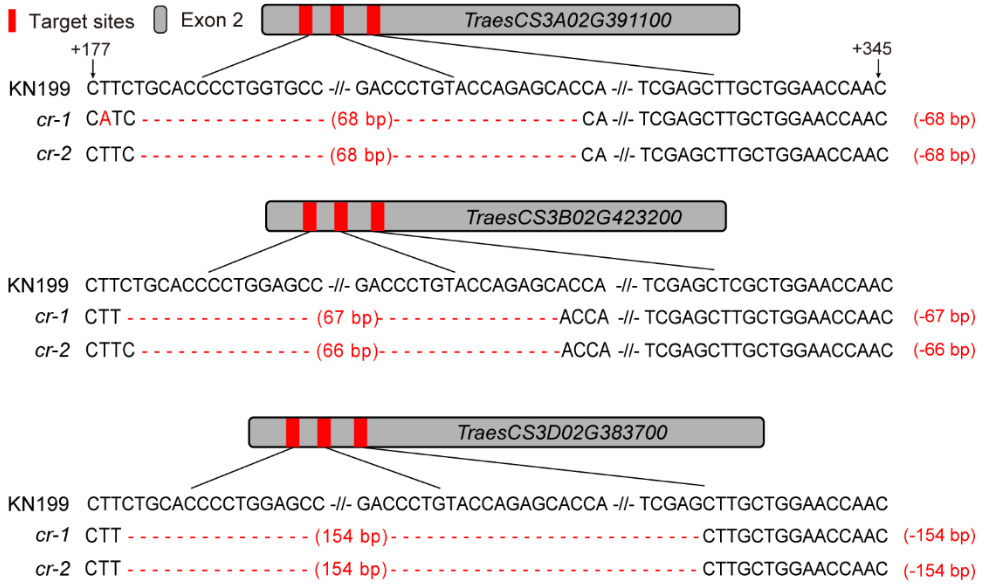

Supplementary Figure 7. Identification of *cr-1* and *cr-2* via sequencing.
Three sgRNA sequences were designed to specifically target exon 2 of the three *HtL1*/*FBA10* homoeologs. Exons are depicted as gray rectangles, and the positions of the target sequences are indicated by red bold lines. The target site sequences for the three sgRNAs and the mutation patterns in the *cr-1* and *cr-2* mutants are shown. Sequencing results highlight a single-nucleotide substitution in red and base deletions as red dashes, with numbers on the right indicating gap lengths.

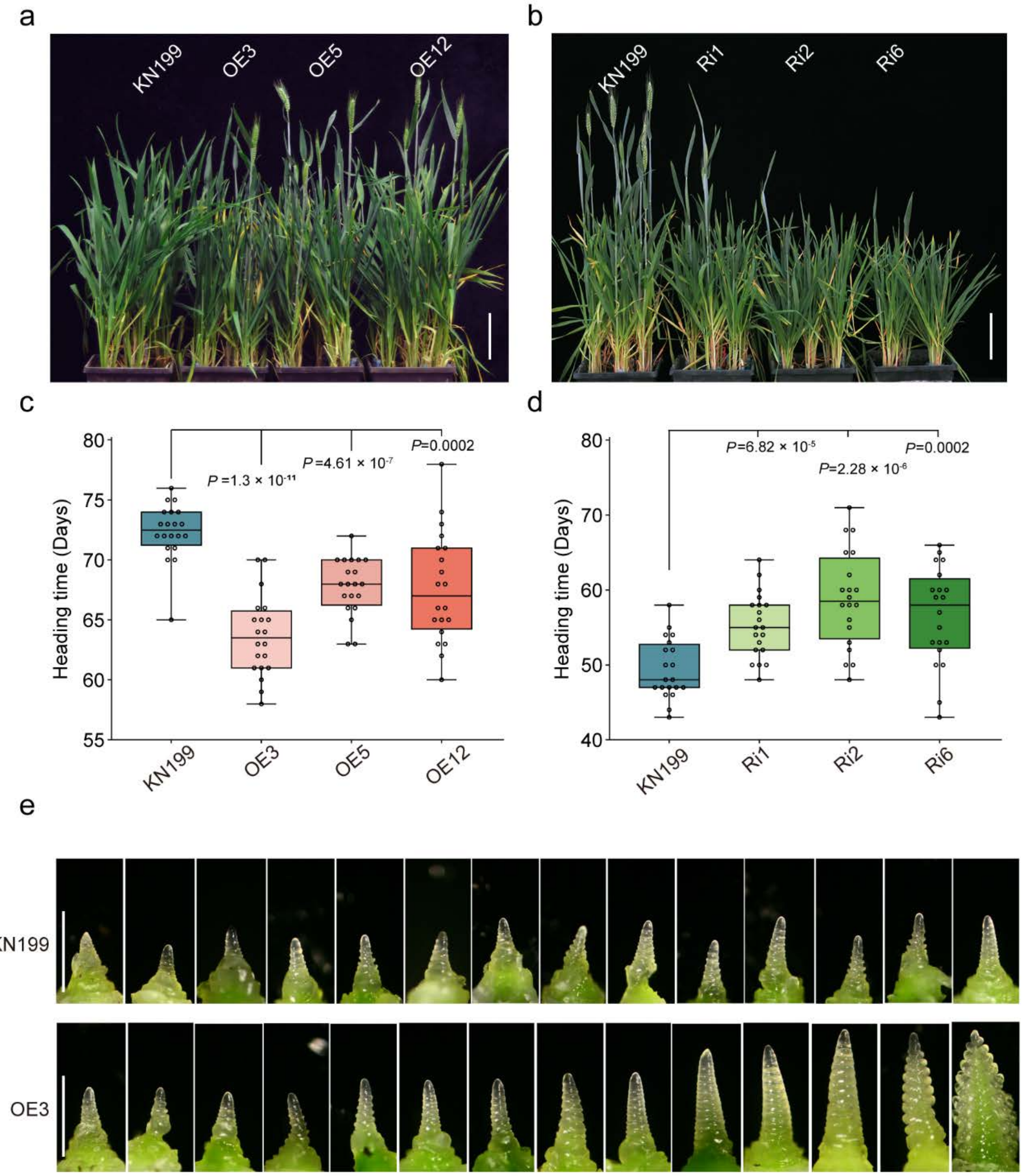

Supplementary Figure 8. *HtL1/FBA10* accelerates flowering in winter wheat.

(a) The morphological phenotype of *HtL1*-OE transgenic wheat plants under 21 days vernalization conditions. Scale bar, 10 cm. **(b)** The morphological phenotype of *HtL1*-RNAi transgenic wheat plants under 28 days vernalization conditions. Scale bar, 10 cm. **(c)** and **(d)** Statistical analysis of phenotypes of heading time of *HtL1*-OE (**c**) and *HtL1*-RNAi lines (**d**). The box plots display the interquartile range, comprising the first quartile, median, and third quartile, while the whiskers extend from the minimum to the maximum values. (n = 20 plants for each group). Two-tailed Student's *t*-test for statistical analysis. The phenotypic observation experiment was performed with three independent biological replicates, yielding consistent results. **(e)** The shoot apical morphology of KN199 and *HtL1*-OE3 plants. Plants were vernalized at 4°C for 30 days, then transplanted in the

green house. After 28 days of growth, a random sample of 14 plants were selected for observation and photographic documentation. Scale bars, 3 mm.

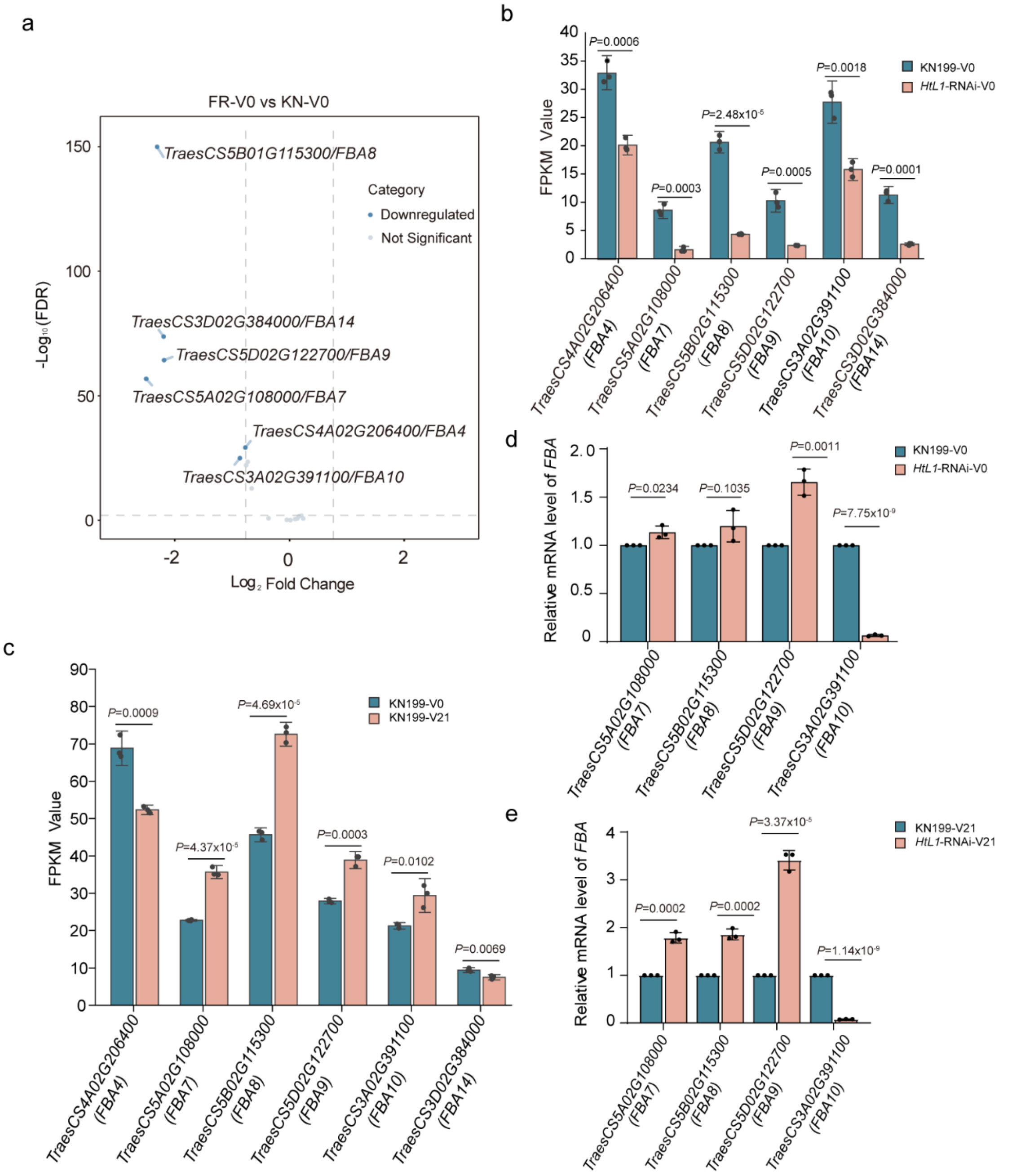

Supplementary Figure 9. Transcription analysis of *FBAs* in *HtL1/FBA10*-RNAi plants.
(a) Volcano plot showing six significantly downregulated *FBA* encoding genes in nonvernalized (V0) *HtL1/FBA10*-RNAi plumules compared to KN199. Nonvernalized wild-type KN199 and *FBA10*-RNAi plumules were collected for Unique Identifier mRNA Sequencing (UID mRNA-seq). FDR<0.01, FR, *HtL1/FBA10*-RNAi. KN, KN199. **(b)** Transcriptional levels analysis of *FBA4*, *FBA7*, *FBA8*, *FBA9*, *HtL1/FBA10*, and *FBA14* indicates a downregulation of these genes, as illustrated in (**a**). Data are means ± SD, two-tailed Student's *t*-test was used for statistical analysis. **(c)** The expression analysis of *FBA4*, *FBA7*, *FBA8*, *FBA9*, *HtL1*/*FBA10* and *FBA14* in response to vernalization in KN199 revealed that, in addition to the upregulation of *HtL1*/*FBA10*, vernalization

also promotes the upregulation of *FBA7*, *FBA8*, and *FBA9*. Plumules of KN199 with (V21) or without (V0) vernalization were collected for UID mRNA-seq. Data are means ± SD, two-tailed Student's *t*-test was used for statistical analysis. **(d)** and **(e)** RT-qPCR analysis showing the relative mRNA expression levels of *FBA7*, *FBA8*, *FBA9*, and *HtL1*/*FBA10* during the stem elongation phase in both KN199 and *HtL1*/*FBA10*-RNAi plants with or without vernalization treatment. These findings indicate a specific knockdown of *HtL1*/*FBA10* in the *HtL1*-RNAi plants. Furthermore, the observed downregulation of *FBA7*, *FBA8*, and *FBA9*, as illustrated in panel (**b**), can be attributed to this particular developmental stage. KN199 and *HtL1/FBA10*-RNAi plants were nonvernalized (V0) (**d**) or vernalized for 21 days (V21) (**e**), respectively. Leaves were collected for RNA extraction, and the transcript levels of indicated *FBAs* were normalized to *ACTIN*, then normalized to KN199 plants. Data are means ± SD, two-tailed Student's *t*-test for statistical analysis (**d** and **e**). All experiments were conducted using three independent biological replicates.

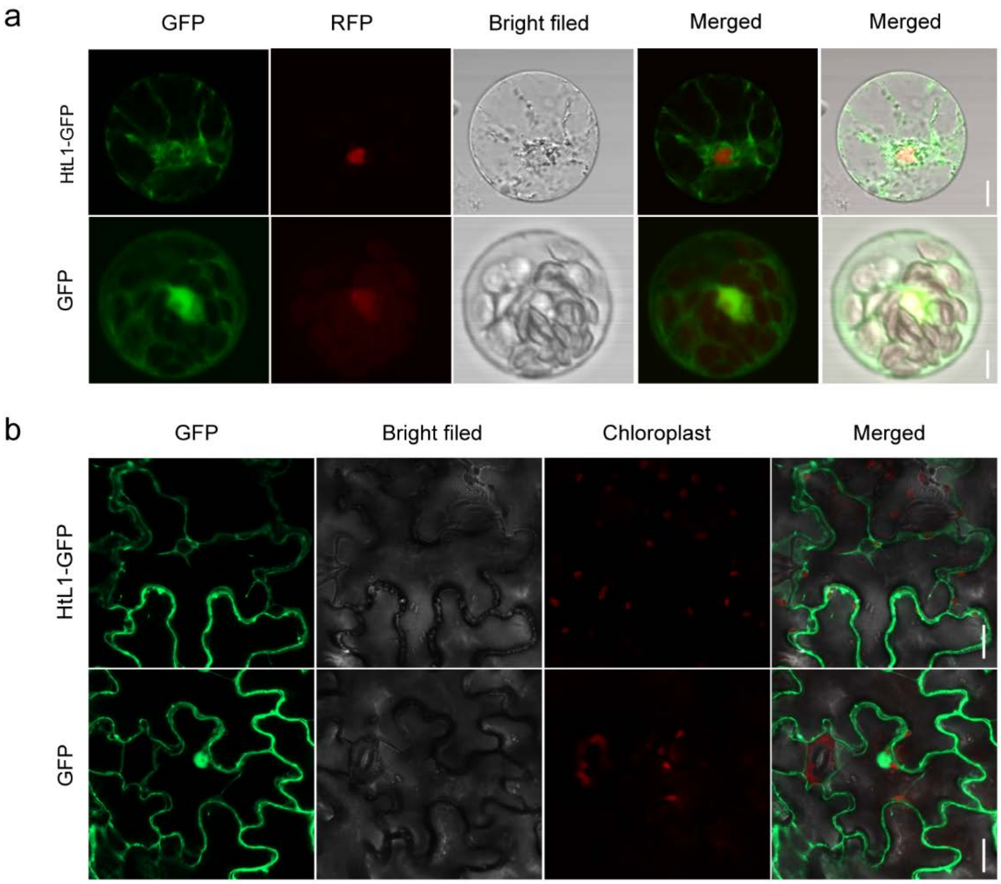

Supplementary Figure 10. The subcellular localization of HtL1-GFP.

(a) The subcellular localization of HtL1-GFP in *Arabidopsis* protoplasts. Scale bars,5 µm. **(b)** The subcellular localization of HtL1-GFP in tobacco leaves. Scale bars, 40 µm. The full-length coding sequence (CDS) of *HtL1* was inserted into the *p*BI221 and *p*BI121 vectors, respectively, and then transformed into *Arabidopsis* protoplasts and *Nicotiana benthamiana* leaves. Confocal laser scanning microscopy was employed to observe the fluorescence signals of GFP.

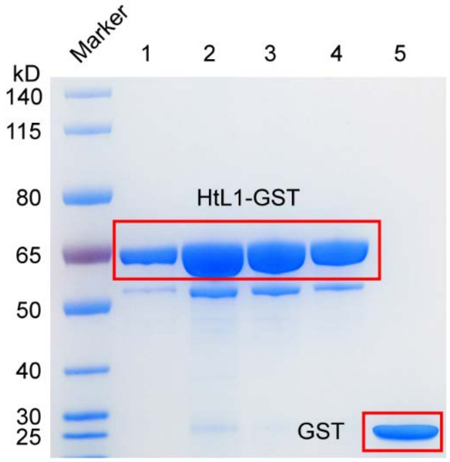

Supplementary Figure 11. **The purification of HtL1-GST and GST *in vitro*.**

The coding sequence (CDS) of *HtL1* was cloned into the *p*ET28a vector, fusion proteins of HtL1-GST and GST expressed in *E. coli* strain BL21 (DE3) were purified. Lane 1-4: Elution fractions 1, 2, 3, and 4 of the purified HtL1-GST fusion protein. Lane 5: First elution fraction of the purified GST protein.

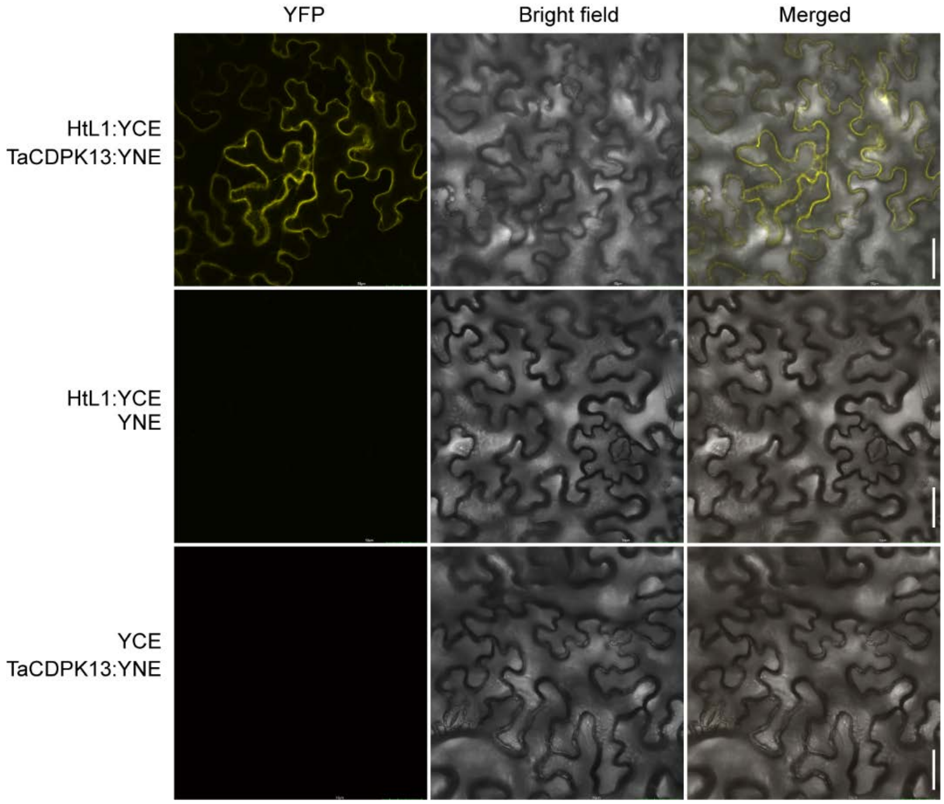

Supplementary Figure 12. BiFC assay of the interaction between HtL1 and TaCDPK13 *in vivo*. The full-length coding sequences of *HtL1* and *TaCDPK13* were inserted into the *p*UC-SPYCE and *p*UC-SPYNE vectors, respectively. Different combinations of constructs were then co-transformed into *Nicotiana benthamiana* leaves. Confocal laser scanning microscopy was employed to observe the fluorescence signals of YFP. Scale bars, 50 μm.

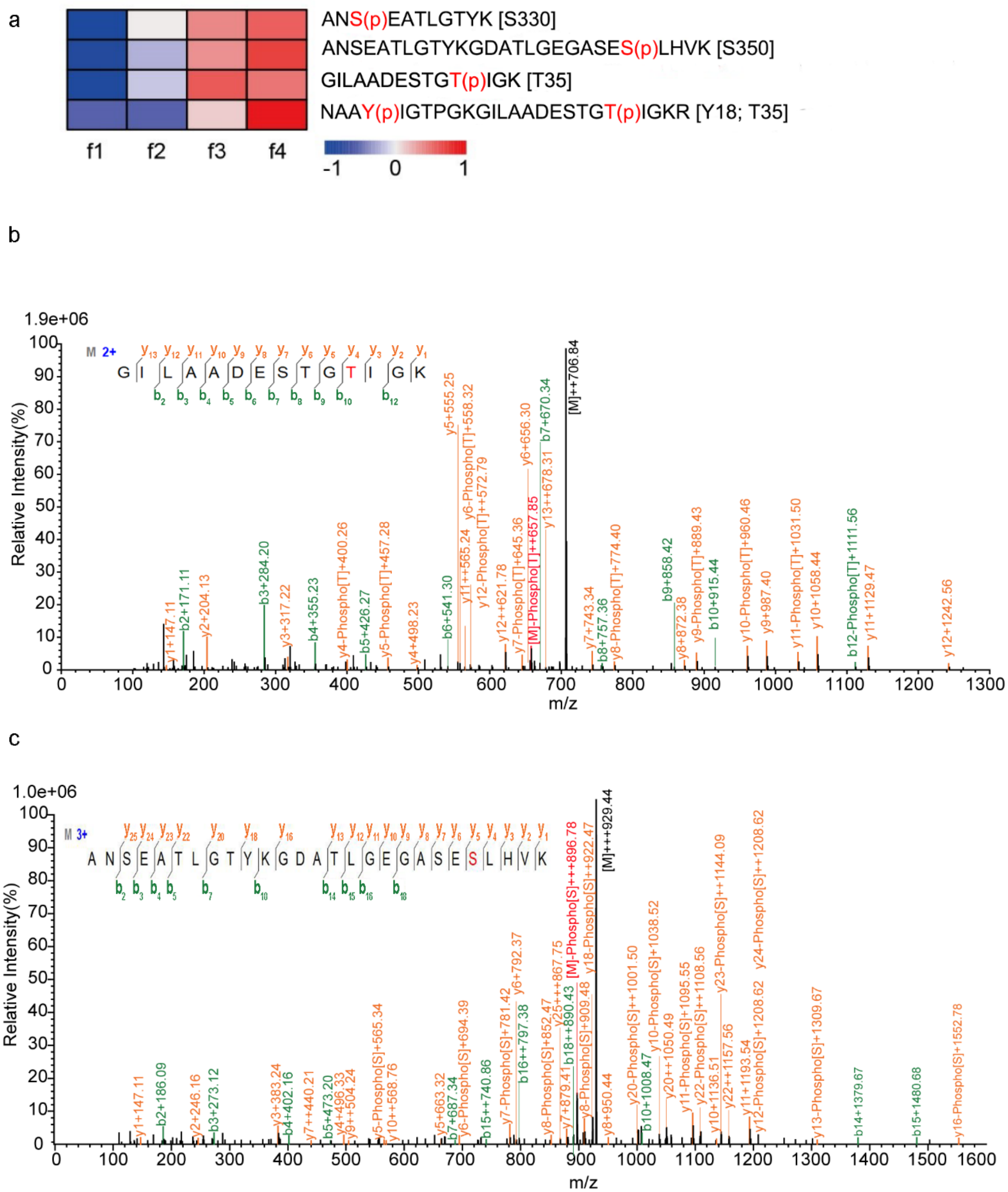

Supplementary Figure 13. Liquid Chromatography with tandem mass spectrometry (LC-MS/MS) identification of phosphorylation at both T35 and S350 on HtL1 expressed in *E. coli*. (a) Detection of variation of HtL1 phosphorylation levels by LC-MS/MS. The HtL1-GST and TaCDPK13-GST fusion proteins were obtained using a prokaryotic expression system, and then purified for *in vitro* phosphorylation assay. The HtL1-GST fusion protein was mixed with different amounts of TaCKPK13-GST, and phosphorylation levels of HtL1 in each reaction were evaluated by mass spectrometry. In f1, no TaCDPK13-GST was added in the action system. From f2 to f4, the amounts of TaCDPK13-GST were gradually increased in the reaction system. By maintaining consistent quantities of HtL1-GST in each reaction, the amounts of four peptides containing

identified phosphorylation sites were observed to increase in response to elevated levels of TaCKPK13-GST within the reaction systems. The color scale represents phosphorylation levels, with blue indicating low levels and red representing high levels. **(b)** Identification of phosphorylation at T35 site on HtL1 using LC-MS. The peptide sequence including the T35 site is GILAADESTGT (35) IGKR. **(c)** Identification of phosphorylation at S350 site on HtL1 using LC-MS/MS. The peptide sequence encompassing the S350 site is represented as ANSEATLGTYKGDATLGEGASES (350) LHVK. Fusion proteins of HtL1-GST and TaCDPK13-GST were expressed and purified *in vitro*, then subjected to *in vitro* phosphorylation reactions, followed by analysis using liquid chromatography-mass spectrometry (LC-MS/MS).

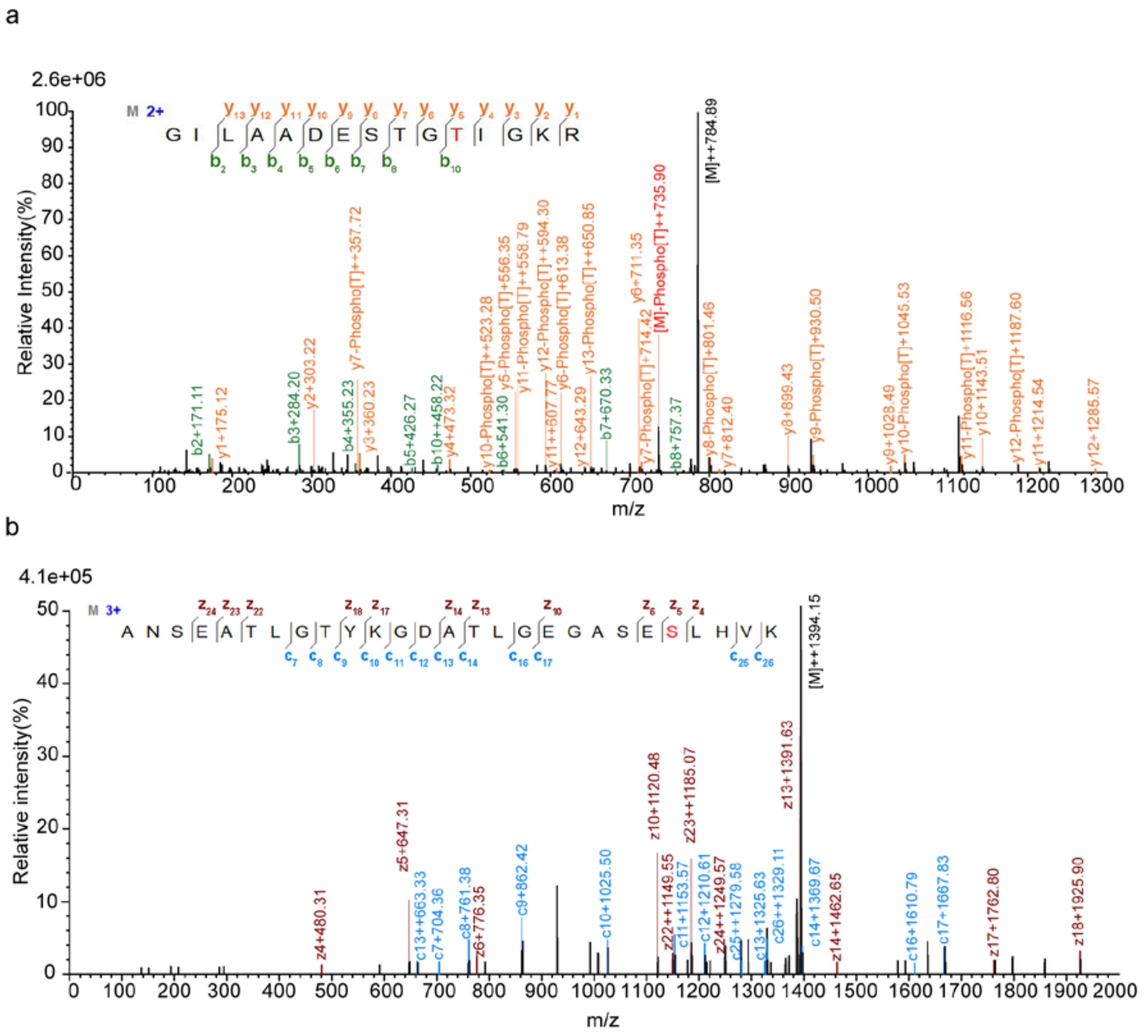

Supplementary Figure 14. Liquid Chromatography with tandem mass spectrometry (LC-MS/MS) identification of phosphorylation at T35 and S350 sites on HtL1 in wheat plants.
(a) Identification of phosphorylation at T35 site on HtL1 using LC-MS/MS. The peptide sequence including the T35 site is GILAADESTGT (35) IGK. **(b)** Identification of phosphorylation modification at S350 site on HtL1 using LC-MS/MS. The peptide sequence including the S350 site is as follows: ANSEATLGTYKGDATLGEGASES (350) LHVK**.** Total proteins were extracted from nonvernalized *HtL1*-OE3 wheat plants. HtL1-HA was immunoprecipitated using an anti-HA antibody, and its phosphorylation modification sites were analyzed by LC-MS/MS.

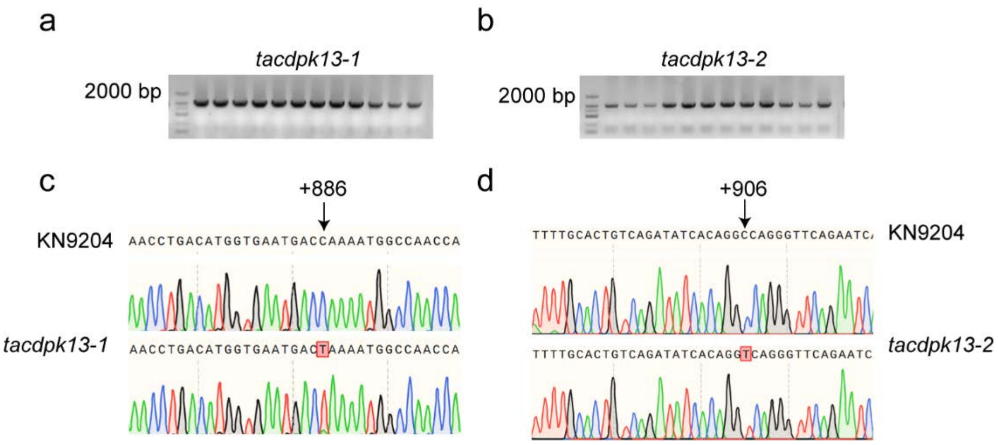

Supplementary Figure 15. Molecular identification of *tacdpk13* mutants.

(a) and **(b)** Identification of *tacdpk13-1* (**a**) and *tacdpk13-2* (**b**) mutants by PCR. **(c)** Sequencing data showing a mutated site in *tacdpk13-1*. **(d)** Sequencing data showing a mutated site in *tacdpk13-2*.

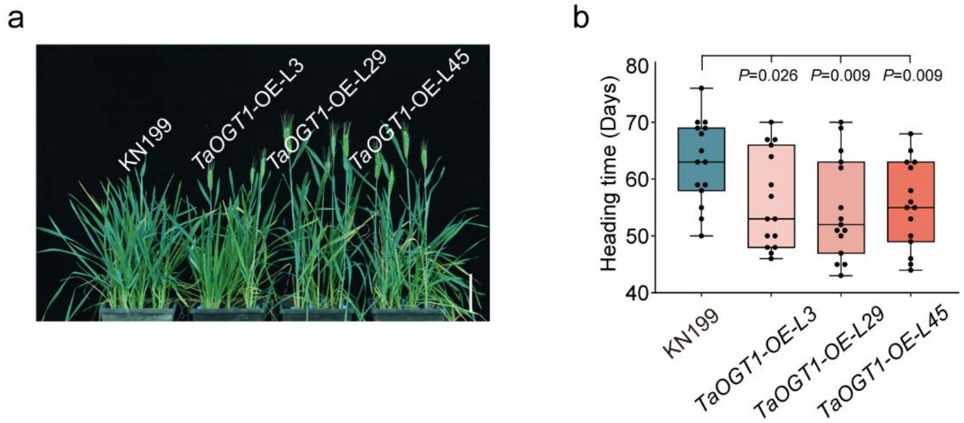

Supplementary Figure 16. *TaOGT1* accelerates flowering in winter wheat.

(a) The morphological phenotype of *TaOGT1*-OE transgenic plants and wild-type KN199 with 28 days vernalization conditions. Scale bar,10 cm. **(b)** Statistical analysis of the heading time of *TaOGT1*-OE lines and KN199. The box plots illustrate the interquartile range, encompassing the first quartile, median, and third quartile, the whiskers extend to the minimum and maximum values. (n=15 plants for each group). Two-tailed Student's *t*-test for statistical analysis. The box plots illustrate the interquartile range, encompassing the first quartile, median, and third quartile, the whiskers extending to represent the minimum and maximum values.

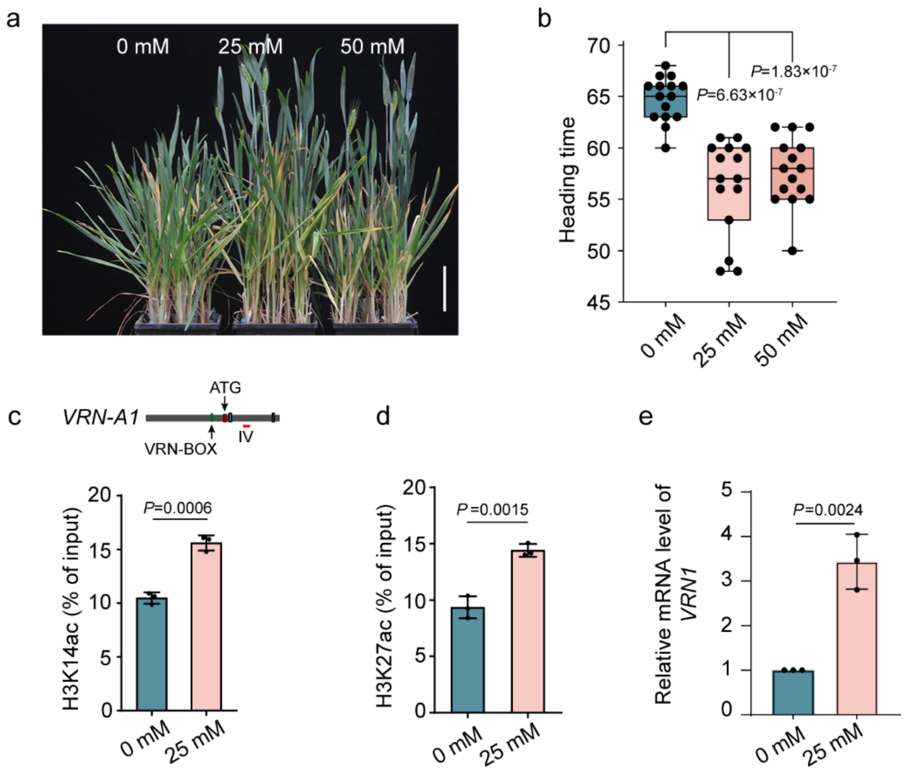

Supplementary Figure 17. FBP treatment enhances H3K14ac and H3K27ac modifications levels at *VRN1*.

(a) Exogenous addition of FBP accelerates flowering in KN199. KN199 was treated with 25 mM and 50 mM FBP during vernalization process, respectively. Wild-type KN199 plants and FBP-treated KN199 plants were transplanted in a green house, following a 21-day vernalization treatment. Scale bar, 10 cm. **(b)** Statistical analyses of heading time of plants in (a). The box plots display the interquartile range, comprising the first quartile, median, and third quartile, while the whiskers extend from the minimum to the maximum values, (n=15 plants for each group). Two-tailed Student's *t*-test for statistical analysis. **(c)** and **(d)** ChIP-qPCR assays of H3K14ac (c) and H3K27ac (d) levels in the region IV at *VRN1*, data are mean ± SD. Schematic diagram of *VRN1*, showing the region detected in ChIP-qPCR analysis. Two independent biological replicates were performed, with each biological replicate comprising three technical replicates. Two-tailed Student's *t*-test for statistical analysis. **(e)** RT-qPCR assay shows that exogenous addition of FBP activates *VRN1* transcription. For (c), (d) and (e), the KN199 plants, 3 days after germination, were subjected to a 21-day vernalization treatment. FBP was applied at 24 hours prior to sampling. Data are means ± SD of three biological replicates. Two-tailed Student's *t*-test was used for statistical analysis.

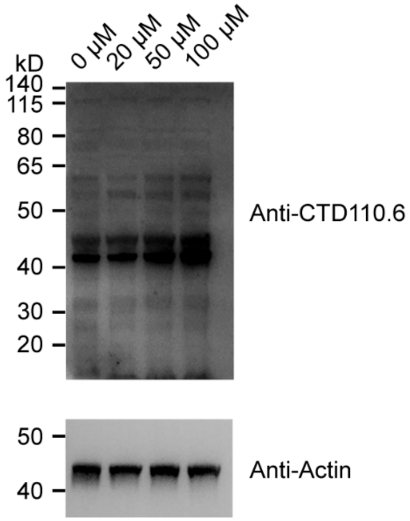

Supplementary Figure 18. Kinase inhibitor KN-93 globally increases *O*-GlcNAc modification of proteins in wheat.

Germinating wheat seeds were treated with the calcium-dependent protein kinase inhibitor KN-93 at concentrations of 0 μM, 20 μM, 50 μM, and 100 μM. Following the extraction of total protein, the levels of *O*-GlcNAc modification were assessed utilizing the anti-CTD110.6 antibody. Actin was probed as the loading control.

Supplementary Method

LC-MS/MS analysis

Fusion proteins of HtL1-GST and TaCDPK13-GST were purified by a prokaryotic expression system and used for in vitro phosphorylation assay. Equal amounts of Ht1L-GST were mixed with increasing amounts of TaCDPK13-GST to create sample groups (n = 2 biological replicates), with Ht1L-GST alone as the negative control (n = 2 biological replicates). To identify *in vivo* phosphorylation of HtL1 in wheat, total proteins from non-vernalized *HtL1*-OE3 plants were immunoprecipitated with an anti-HA antibody, and the enriched HtL1-HA sample was analyzed by LC-MS/MS (n = 2 biological replicates).

To digest protein, incubate the sample with 5mM TCEP at 55°C for 30 minutes, then add 25mM iodoacetamide and incubate in the dark at room temperature for 30 minutes. Finally, add 1μg trypsin and incubate at 37°C for 16 hours. For the LC-MS/MS analysis, the samples were dissolved in Solvent A, comprising 0.1% formic acid in water, and subsequently introduced into a manually packed reverse-phase C18 column (25 cm in length, 150 μm inner diameter), featuring C18 resin with a particle size of 1.9 μm and a pore diameter of 120 Å, supplied by Dr. Maisch GmbH Inc., Germany. This column was integrated with an Easy nLC-1200 nanoLC system (Thermo Fisher Scientific, Waltham, MA). The elution of samples was performed using a 75-minute gradient at a flow rate of 600 nL/min, starting with 7% and increasing to 12% of Solvent B, which consists of 0.1% formic acid and 20% water in acetonitrile, over a period of 7 minutes, 12% to 30% solvent B over 45 minutes, 30% to 40% solvent B over 10 minute, 40% to 90% solvent B over 1 minute, and 90% solvent B held for 12 minutes. A Thermo Orbitrap Fusion Lumos mass spectrometer (Thermo Fisher Scientific) was employed for sample analysis using an HCD-pd-EThcD method with the following parameters: the resolution was set to 120,000 for MS1, where the scan range was 350–1800 m/z. The automatic gain control (AGC) target was 4×10^5, and the selected charge states were 2–8. Dynamic exclusion was set to 30 seconds with an exclusion window of 10 ppm and a cycle time of 3 seconds.

For HCD MS/MS, the resolution was set to 15,000. Ions were fragmented via higher collision dissociation and analyzed by the Orbitrap with an AGC target of 5×10^4 or a maximum injection time of 90 ms. The collision energy was set to a stepped mode of 20%, 30%, and 40%. When signature MS/MS oxonium ions of phosphorylation (32.6589 m/z, 48.9884 m/z, 97.9768 m/z) were detected, EThcD MS/MS on the same precursor was triggered (isolation window of 1.6 m/z). Fragment ions were analyzed in the Orbitrap mass analyzer at a resolution of 30,000, with an AGC target value of 1×10^5 or a maximum injection time of 90 ms, using EThcD activation and supplemental activation with a normalized collision energy of 30%.

For MS data analysis, identification and LFQ analysis was performed on Proteome Discoverer (version 2.4) software, and the Sequest search engine was used for searching raw data against protein database. Raw data from in vivo wheat samples were searched against the *Triticum aestivum* database from IWGSC, using *Arabidopsis thaliana* protein annotations. Raw data from in vitro purified samples were compared with both the *Triticum aestivum* and UniProt *E. coli* ver.20230612 databases. In the analysis of proteomic data, the precursor mass tolerance was configured to 20 parts per million (ppm), while the fragment mass tolerance was established at 0.05 Daltons (Da). Trypsin/p was employed as the enzyme, allowing for a maximum of two missed cleavages. Carbamidomethylation of cysteine residues (C) was designated as a fixed modification, whereas oxidation of methionine (M), phosphorylation of serine, threonine, and tyrosine (S/T/Y),

and acetylation at the protein N-terminus were specified as variable modifications across all software programs utilized. The peptide length was constrained to a minimum of 6 amino acids and a maximum of 144 amino acids. The rest of the parameters were set as default.

For the label-free quantitative (LFQ) proteomics analysis, normalization and scaling modes were applied with the "total peptide amount" setting. Proteins were retained for subsequent analysis only if they met two criteria: a "High" confidence level in the Protein FDR Confidence Combined and a "Master" designation as "IsMasterProtein". The built-in t-test (Background Based) of the Proteome Discoverer software was used for quantitation. Quantitative results with an adjusted *P*-value (abundance ratio adj. *P*-value) < 0.05 were adopted. All other parameters were set as default.
