# Supplementary material for: O-GlcNAc and phosphorylation modifications on HtL1/FBA10 regulate wheat vernalization for flowering
Source: Nat Commun. 2025 Dec 27;17:999. doi: 10.1038/s41467-025-67734-0 (PMC12848038; doi:10.1038/s41467-025-67734-0)
Supplement: Supplementary file 8 — Reporting Summary [file 41467_2025_67734_MOESM8_ESM.pdf]

nature portfolio

Corresponding author(s): Kang Chong

Last updated by author(s): Oct 16, 2025

Reporting Summary

Nature Portfolio wishes to improve the reproducibility of the work that we publish. This form provides structure for consistency and transparency in reporting. For further information on Nature Portfolio policies, see our Editorial Policies and the Editorial Policy Checklist.

Statistics

For all statistical analyses, confirm that the following items are present in the figure legend, table legend, main text, or Methods section.

n/a	Confirmed	
☐	☒	The exact sample size (*n*) for each experimental group/condition, given as a discrete number and unit of measurement
☐	☒	A statement on whether measurements were taken from distinct samples or whether the same sample was measured repeatedly
☐	☒	The statistical test(s) used AND whether they are one- or two-sided<br>*Only common tests should be described solely by name; describe more complex techniques in the Methods section.*
☒	☐	A description of all covariates tested
☐	☒	A description of any assumptions or corrections, such as tests of normality and adjustment for multiple comparisons
☐	☒	A full description of the statistical parameters including central tendency (e.g. means) or other basic estimates (e.g. regression coefficient) AND variation (e.g. standard deviation) or associated estimates of uncertainty (e.g. confidence intervals)
☐	☒	For null hypothesis testing, the test statistic (e.g. *F*, *t*, *r*) with confidence intervals, effect sizes, degrees of freedom and *P* value noted<br>*Give P values as exact values whenever suitable.*
☒	☐	For Bayesian analysis, information on the choice of priors and Markov chain Monte Carlo settings
☒	☐	For hierarchical and complex designs, identification of the appropriate level for tests and full reporting of outcomes
☒	☐	Estimates of effect sizes (e.g. Cohen's *d*, Pearson's *r*), indicating how they were calculated

Our web collection on statistics for biologists contains articles on many of the points above.

Software and code

Policy information about availability of computer code

Data collection	The genotypic data were not directly collected in this study but were derived from the research titled "Resequencing of 145 landmark cultivars reveals asymmetric sub-genome selection and strong founder genotype effects on wheat breeding in China," published by Xueyong Zhang et al. in Molecular Plant (2022, Volume 13, Issue 12, Pages 1733-1751). We obtained these data by requesting them directly from the original authors and receiving their consent.<br>Leica TCS SP5 confocal microscope was used to capture images in BiFC assay and subcellular localization assays.<br>CCD camera (Tanon-5200) were used to expose and capture images in western blot.<br>Quant Studio3 (Thermo Fisher) was used in qPCR.<br>Two-dimensional liquid chromatography–high-resolution tandem mass spectrometry is used for the identification of phosphorylation sites.
Data analysis	The GWAS was performed for heading time traits on 91 wheat accessions by GEMMA (Genome-wide Efficient Mixed Model Association) software.<br>Image J was used to analyze band intensity of pictures in western blot .<br>Two-tailed Student’s t-test for statistical analysis.<br>Adobe Photoshop 2022 was used to cut images from western blot.

For manuscripts utilizing custom algorithms or software that are central to the research but not yet described in published literature, software must be made available to editors and reviewers. We strongly encourage code deposition in a community repository (e.g. GitHub). See the Nature Portfolio guidelines for submitting code & software for further information.

Data

Policy information about availability of data

All manuscripts must include a data availability statement. This statement should provide the following information, where applicable:

- Accession codes, unique identifiers, or web links for publicly available datasets
- A description of any restrictions on data availability
- For clinical datasets or third party data, please ensure that the statement adheres to our policy

Provide your data availability statement here.

Research involving human participants, their data, or biological material

Policy information about studies with human participants or human data. See also policy information about sex, gender (identity/presentation), and sexual orientation and race, ethnicity and racism.

Reporting on sex and gender	No sex and gender need to be considered in the study.
Reporting on race, ethnicity, or other socially relevant groupings	No race, ethnicity, or other socially relevant groupings need to be considered in the study.
Population characteristics	No population characteristics need to be considered in the study.
Recruitment	No participants were recruited.
Ethics oversight	This study does not involve ethics insight.

Note that full information on the approval of the study protocol must also be provided in the manuscript.

Field-specific reporting

Please select the one below that is the best fit for your research. If you are not sure, read the appropriate sections before making your selection.

[x] Life sciences [ ] Behavioural & social sciences [ ] Ecological, evolutionary & environmental sciences

For a reference copy of the document with all sections, see nature.com/documents/nr-reporting-summary-flat.pdf

Life sciences study design

All studies must disclose on these points even when the disclosure is negative.

Sample size	No statistical methods were used to predetermine sample size. For wheat flowering phenotype observation, at least 12 wheat plants were used. For protein extraction, immunoblotting and RNA isolation, at least five plants were collected per sample to minimize individual variation.
Data exclusions	No data were excluded from the analyses.
Replication	we confirm that three independent biological replicates were performed. All attempts at replication were successful.
Randomization	In the plant experiments of this study, we employed a systematic random sampling strategy. Specifically, from a population of plants with uniform genotypes, synchronized sowing, and controlled growth conditions, All selected samples were at the same developmental stage to ensure comparability between samples. This sampling approach effectively avoided selection bias and ensured the representativeness of the samples. In the comparative analysis of transgenic plants and wild-type, grouping is based on genotype rather than random assignment. To ensure experimental comparability, wild-type and mutant seeds are harvested simultaneously, their germination is synchronized, and they are cultivated under identical environmental conditions. Consequently, any observed differences can be attributed to genotypic variations.
Blinding	Blinding was not applicable as all data were collected through quantitative instrumentation and genetic backgrounds were known for molecular analyses.

Reporting for specific materials, systems and methods

We require information from authors about some types of materials, experimental systems and methods used in many studies. Here, indicate whether each material, system or method listed is relevant to your study. If you are not sure if a list item applies to your research, read the appropriate section before selecting a response.

Materials & experimental systems

n/a	Involved in the study
☐	☒ Antibodies
☒	☐ Eukaryotic cell lines
☒	☐ Palaeontology and archaeology
☒	☐ Animals and other organisms
☒	☐ Clinical data
☒	☐ Dual use research of concern
☐	☒ Plants

Methods

n/a	Involved in the study
☒	☐ ChIP-seq
☒	☐ Flow cytometry
☒	☐ MRI-based neuroimaging

Antibodies

Antibodies used

Anti-HA(Sigma, H9658), Anti-Actin(Huaxingbio,HX1843), Anti-Phospho - (Ser/Thr) Phe(abcam,ab17464), Anti-CTD110.6 (Cell Signaling Technology, 9875S), Anti-H3(Abcam, ab1791), Anti-H3K27ac(Abcam, ab4729), Anti-H3K14ac(Abcam,Ab52946), Anti-H3K9ac (Abcam,ab218553), Anti-H3K4me3(Millipore, 07473). Secondary antibody (Cell Signaling Technology, anti-rabbit lgG, 7074), Secondary antibody (Cell Signaling Technology, anti-mouse lgG, 7076).

Validation

For the commercial antibody, all validation statements can be found on the manufacturer's website.

Plants

Seed stocks

All experimental seeds were maintained in the laboratory's wheat germplasm resources, except for the Tacdpk13 mutant, which was acquired from the KN9204 mutant collection.

Novel plant genotypes

The HtL1-OE plant was generated by using the constitutive ubiquitin promoter in the winter cultivar KN199.
HtL1 knockdown mutants using RNA interference (RNAi) technology, and a specific 273 bp CDS sequence of HtL1 was selected for precise targeting.

Authentication

The HtL1-overexpressing transgenic lines were molecularly characterized, confirming successful gene overexpression in planta. Based on Unique Identifier mRNA Sequencing (UID mRNA-seq) data derived from wheat plumules of KN199 and HtL1-RNAi plants without vernalization treatment, as well as further qRT-PCR analysis of indicated genes, we evaluated the impact of HtL1/FBA10 knockdown on the transcription of other homologous genes, indicating that HtL1 knockdown achieved the anticipated specificity.
