# Supplementary material for: O-GlcNAc and phosphorylation modifications on HtL1/FBA10 regulate wheat vernalization for flowering
Source: Nat Commun. 2025 Dec 27;17:999. doi: 10.1038/s41467-025-67734-0 (PMC12848038; doi:10.1038/s41467-025-67734-0)
Supplement: Supplementary file 9 — Description of Additional Supplementary Files [file 41467_2025_67734_MOESM9_ESM.pdf]

Description of Additional Supplementary Files

Supplementary Data 1. Information of accessions used in this study.

Supplementary Data 2. The expression of candidate genes in interval.

Supplementary Data 3. Natural variation of candidate genes in interval.

Supplementary Data 4. Detailed information on SNP loci within 2 kb upstream and downstream of the *TraesCS3A02G391100* gene.

Supplementary Data 5. The potential interaction proteins of HtL1 identified by IP-MS.

Supplementary Data 6. List of primers used in this study.
